# Improving Antimicrobial Properties of GelMA Biocomposite Hydrogels for Regenerative Endodontic Treatment

**DOI:** 10.3390/polym16121675

**Published:** 2024-06-12

**Authors:** Ozgul C. Dorterler, Berre Akgun, Mehlika Alper, Fatma Ayhan

**Affiliations:** 1 Department of Pediatric Dentistry, Faculty of Dentistry, Muğla Sıtkı Koçman University, Muğla 48000, Türkiye; ozgulcarti@mu.edu.tr; 2 Department of Molecular Biology and Genetics, Faculty of Science, Muğla Sıtkı Koçman University, Muğla 48000, Türkiye; berreakgun@posta.mu.edu.tr (B.A.); mehlikaalper@mu.edu.tr (M.A.); 3 Biochemistry & Biomaterials Research Group (BIOMATREG), Department of Chemistry, Biochemistry Division, Faculty of Science, Muğla Sıtkı Koçman University, Muğla 48000, Türkiye

**Keywords:** biomaterial, biocomposite hydrogels, regenerative endodontic treatment

## Abstract

Regenerative endodontics is a developing field involving the restoration of tooth structure and re-vitality of necrotic pulp. One of the most critical clinical considerations for regenerative endodontic procedures is the disinfection of the root canal system, since infection interferes with regeneration, repair, and stem cell activity. In this study, we aimed to provide the synthesis of injectable biopolymeric tissue scaffolds that can be used in routine clinical and regenerative endodontic treatment procedures using Gelatin methacryloyl (GelMA), and to test the antimicrobial efficacy of Gelatin methacryloyl/Silver nanoparticles (GelMA/AgNP), Gelatin methacryloyl/Hyaluronic acid (GelMA/HYA), and Gelatin methacryloyl/hydroxyapatite (GelMA/HA) composite hydrogels against microorganisms that are often encountered in stubborn infections in endodontic microbiology. Injectable biocomposite hydrogels exhibiting effective antimicrobial activity and non-cytotoxic behavior were successfully synthesized. This is also promising for clinical applications of regenerative endodontic procedures with hydrogels, which are proposed based on the collected data. The GelMA hydrogel loaded with hyaluronic acid showed the highest efficacy against *Enterococcus faecalis*, one of the stubborn bacteria in the root canal. The GelMA hydrogel loaded with hydroxyapatite also showed a significant effect against *Candida albicans*, which is another bacteria responsible for stubborn infections in the root canal.

## 1. Introduction

Tissue engineering is a multidisciplinary field that combines the principles of engineering, biochemistry, biology, and clinical sciences to develop biological models that can maintain and improve the function of organs and tissues [1]. In this context, regenerative endodontics is a developing field involving the restoration of tooth structure and re-vitality of necrotic pulp [2].

Recently, hydrogel-based scaffolds have been introduced into the field of tissue engineering. It is a unique category of three-dimensional (3D) polymeric networks containing water as a liquid component. Their hydrophilic structure allows them to retain a high water content and biological fluids, as well as the diffusion of nutrients through their structure. In addition to their biocompatibility, expected degradation patterns, and adjustable mechanical properties, they can maintain their network integrity, therefore, they are insoluble at high water concentrations due to their crosslinked structure.

Furthermore, hydrogels have a significant degree of flexibility and elasticity, similar to a natural extra-cellular matrix (ECM), which provides the essential cell support needed during tissue regeneration. Therefore, in addition to their gelatinous structure and the fact that they can be loaded with different drugs, they are considered to be an optimal choice for many tissue engineering applications due to these unique properties [3,4].

At the biological level, hydrogel building scaffolds are used for dentin–pulp complex regeneration, and their degradation products should be biocompatible, non-toxic, non-immunogenic, and should not cause significant inflammatory reactions. They should also allow for the greatest encapsulation of cells and support the adhesion of cells to the surface and cellular migration, proliferation, differentiation, and function [5,6].

Regenerative endodontic procedures (REPSs) are defined as “biologically based procedures designed to replace damaged structures, including dentin and root structures, as well as cells of the pulp–dentin complex” [2]. One of the most critical clinical considerations for REPSs is the disinfection of the root canal system, since infection interferes with regeneration, repair, and stem cell activity [7]. Chemical disinfection of the root canal system depends not only on the bactericidal/bacteriostatic properties of antibacterial agents, but also on the fact that these irrigants/medicines should not damage the survival and proliferation capacity of the patient’s stem cells [8].

According to current concepts in endodontic microbiology, the resulting infection is a biofilm-mediated infection, which is considered to be the most stubborn type of chronic root canal infection [9,10]. Although adequate antimicrobial efficacy is one of the main advantages of the use of topical antibiotic pats, clinical limitations have emerged [11]. The complete removal of the applied antibiotic pat is crucial for a successful regenerative outcome and to avoid unwanted, possibly long-term side effects. Unfortunately, studies have shown that a significant amount of antibiotic pat (88% residue) remains in the root canal system after current irrigation techniques [12]. Indirect negative effects such as a decrease in dentin strength and fracture resistance have been reported 1 week after application. These effects are mainly related to the strong demineralization effect and acidic structure of antibiotic pats [13,14]. There are contradictions regarding the antimicrobial efficacy of Ca(OH)_2_, another agent used in the disinfection of the root canal system [11]. The ability of Ca(OH)_2_ to eliminate specific bacteria such as *Enterococus faecalis* from root canal systems has been questioned [15]. From a biological point of view, Ca(OH)_2_ provides a favorable environment for stem cell survival and proliferation [16]. However, the possible side effects of Ca(OH)_2_ on the biological properties of dentine-matrix-derived growth factors have been highlighted and should be taken into account [17].

Gelatin is a natural collagen derivative obtained through the hydrolysis of the collagen triple helix into single molecules through alkaline- or acid-processing processes [18]. Gelatin is biocompatible, natural, hydrophilic, biodegradable, and non-immunogenic [19,20]. GelMA hydrogels are usually obtained by reacting methacrylic anhydride with gelatin and optically cross-linking it in the presence of a photoinitiator [21,22]. Unlike other existing hydrogel-forming biomaterials, GelMA hydrogels can be made to meet the biological functionality and mechanical adjustability requirements of most tissue engineering applications by adapting the synthetic process or by adding various biomaterials. GelMA hydrogels combine the advantages of natural and synthetic hydrogels and are widely used in tissue engineering applications [23]. GelMA hydrogels are widely used for various tissue engineering applications [22], but the impression obtained from the research conducted in the literature is that very little has been researched about the dental pulp regeneration applications of these scaffold materials.

Silver nanoparticles (AgNPs) have been proposed as an antibacterial agent for endodontic disinfection due to their broad-spectrum antibacterial properties and lesser effects on inducing microbial resistance compared to antibiotics [24,25]. Silver is the most widely used metal nanomaterial for inhibiting various types of microorganisms and drug-resistant microorganisms [26]. AgNP is known to provide certain benefits in dentistry, especially in endodontic treatment, due to its important antibacterial effects against both Gram-negative and Gram-positive pathogens [27].

The main inorganic component of bone and enamel is hydroxyapatite (HA). The addition of bioactive elements as fillers to tissue scaffolds can improve their physical and chemical properties; therefore, scaffolds containing inorganic and polymeric materials can increase mechanical strength, biocompatibility, and biodegradability [28]. Small nanoHA particles (<100 nm) have been reported to be more suitable for the host due to improved adhesion, cell proliferation, and cell adhesion [29].

In addition, Ragap et al. [30]. reported that hydroxyapatite nanoparticles are active against the most common Gram-positive and Gram-negative bacteria and are an advantageous material for clinical applications.

Hyaluronic acid (HYA) is an unsulfated glycosaminoglycan found in the ECM of many soft connective tissues [27]. HYA is biocompatible, biodegradable, bioactive, immunogenic, and non-thrombogenic, and has a high water affinity [31]. HYA was recently investigated to evaluate its bacteriostatic properties and showed dose-dependent effects on different microorganisms [32]. Antimicrobial studies using Gram-positive bacteria from streptococcus and enterococcus species have shown that HYA has dose-dependent bacteriostatic effects against *Streptococcus* ATCC 25175, *Enterococcus faecalis* ATCC 29212, and *Enterococcus hirae* ATCC 10541 [31]. A porous tissue engineering scaffold produced from HYA showed that *E. coli* (ATCC 11229) has bacteriostatic effects by reducing colony formation units (CFU) from 3.9 × 10^7^ (control) to 50 (with HYA) [33].

There are a limited number of studies in the literature where AgNPs, HA [34], and HYA [35] have been used as biocomposite injectable hydrogels with GelMA. However, there have been no studies conducted on the success of antimicrobial activity against *Enterococcus faecalis* and *Candida albicans*, the main microorganisms responsible for the failures that occur during endodontic treatments.

In addition, the main innovation we are aiming for in this study is to eliminate the disadvantages of using Ca(OH)_2_ or the antibiotic pat used in routine treatment by increasing the antimicrobial effectiveness of the hydrogels to be synthesized. This limited antimicrobial effect may lead to the elimination of residual paste, bacterial resistance. Dentin durability and the number of treatment sessions may also decrease.

In this study, we aimed to provide the synthesis of injectable biopolymeric hydrogel scaffolds that can be used in routine clinical treatment and regenerative endodontic treatment procedures. For this purpose, we invetigated swelling behavior, the cellular viability of L929 cells, and the antimicrobial properties against the microorganisms that are often encountered in stubborn infections in endodontic microbiology by synthesizing GelMA, GelMA/AgNP, GelMA/HA, and GelMA/HYA composite hydrogels.

## 2. Materials and Methods

### 2.1. GelMA Synthesis

GelMA synthesis: Type B 225 bloom (40,000–50,000 Da) (Sigma Aldrich, St. Louis, MI, USA) gelatin was used. A solution was prepared with 0.1 M carbonate/bicarbonate buffer (Merck, Germany) to be 10% (*w*/*v*) gelatin. This solution was dissolved by stirring at 50 °C for 30 min. After the gelatin was dissolved, heating was continued and methacrylic anhydride (MAA) (Sigma Aldrich, St. Louis, MI, USA) was added at a rate of 0.1 mL per 1 g of gelatin. After the addition of methacrylic anhydride (MAA) was completed, the reaction was continued by stirring for another half hour. The solution obtained after the reaction was transferred into dialysis bags and dialyzed at 37 °C. The GelMA was obtained after the dialysis was lyophilized and stored at −20 °C [36,37].

**Figure 1:**
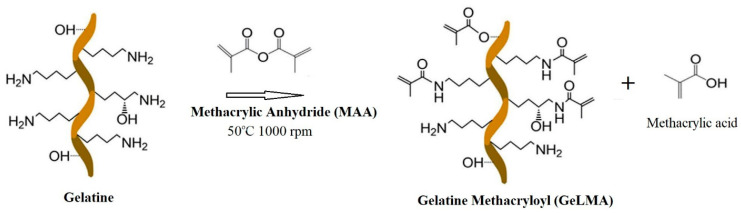
Schematic representation of the synthesis of GelMA.

The synthesis of GelMA occurred by the reaction of gelatin and methacrylic anhydride. The reaction of the Ɛ-amino (-NH_3_) groups contained in gelatin with methacrylic anhydride resulted in gelatin methacryloyl and methacrylic acid (Figure 1) in the environment. Dialysis was performed in order to remove the methacrylic acid occurring in the environment.

### 2.2. Determination of the Degree of Substitution of GelMA

The TNBS (2,4,6-trinitrobenzene-1-sulfonic acid) method was used to determine the degree of GelMA substitution. Using the TNBS method, a calibration graph was prepared with glycine. Depending on the obtained graph, the degree of substitution of the synthesized GelMA was determined. In the TNBS analysis, glycine solutions in different concentrations were prepared, while the calibration graph was prepared with glycine. In total, 0.5 mL of 0.01% TNBS solution was added to 0.5 mL of standard solution. The obtained mixture was incubated at 37 °C for 2 h and the results were evaluated by reading the absorbance at 335 nm [37].

### 2.3. Injectable GelMA Biocomposite Hydrogel Synthesis

The synthesized GelMA (10%) was heated in ultrapure water and the solution was prepared. Pure GELMA hydrogel was synthesized by adding photoinitiating Irgacure 2959 (2-Hydroxy-4’-(2-hydroxyethoxy)-2-methylpropiophenone) at a rate of 1% to this solution and photopolymerizing with a UV light source at a wavelength of 365 nm. GelMA (10%) for the GelMA/HA hydrogel synthesis was obtained by photopolymerization by adding Irgacure at a rate of 1% to hydroxyapatite solution prepared to be 1 mg/mL. GelMA (10%) for the GelMA/HYA hydrogel synthesis was obtained by photopolymerization by adding Irgacure at a rate of 1% in hyaluronic acid solution prepared to be 1 mg/mL. In the GelMA/AgNP hydrogel synthesis, the AgNP solution was first sonicated [38,39]. After this, it was obtained by photopolymerization by adding GelMA and Irgacure to this solution.

### 2.4. GelMA and GelMA Hydrogel Characterization

In the characterization of the synthesized GelMA and the resulting GelMA hydrogel, FTIR (Fourier Transform Infrared Spectrophotometry) was used to determine the functional groups found in the mass and surface structures. H-NMR (Nuclear Magnetic Resonance Spectrometry) was used to determine the structure of an atom or molecule within a structure. An optical microscope was used for preliminary visual morphological determination with SEM (Scanning Electron Microscope, Scanning Electron Microscope), a determination of the morphologies and pore structures of hydrogels, and a determination of the swelling values of microsystems.

### 2.5. AgNP Synthesis (Green Synthesis)

In September, 0.15 g of collected, washed, and dried *Liquidambar orientalis* Mill. leaves was weighed and thrown into 150 mL of bidistilled pure water, which reached boiling point, and then boiled for 20 min at 100 °C in a magnetic heater. After cooling the solution at room temperature, it was filtered with the help of a coarse filter. In the second stage, 1 mL of silver nitrate (AgNO_3_) and 89 mL of ultra-pure water were added to 10 mL of extract and a 100 mL solution was prepared. The prepared solution was centrifuged in 1.5 µL Eppendorf tubes in a refrigerated centrifuge at 14,000 rpm/20,000 g for 1 h. The supernatants were taken from the Eppendorf tubes, 500 µL of ultrapure water was added to the sediments, and a sonication process was applied for 5 h.

### 2.6. Cell Culture and Cell Viability Assays

Hydrogel synthesis was performed in laminar flow hood using sterilized materials and equipment. The resulting hydrogel structure was swollen in PBS in an incubator at 37 °C to simulate the physiological pH and temperature conditions overnight and was used in the cell experiments. DMEM medium supplemented with 10% heat-inactivated FBS and penicillin–streptomycin (100–100 μg/mL) antibiotic was used to culture L929 mouse fibroblast cells. The cells were incubated at 37 °C in a CO_2_ incubator supplied with 5% CO_2_ and 95% humidity.

The viability of the L929 cells treated with injectable hydrogel structures was determined by 3-(4,5-Dimethylthiazol-2-yl)-2,5-Diphenyltetrazolium Bromide (MTT) assay with some modifications for 24 h [40,41]. The cells were first applied to 96-well plates at a density of 2 × 10^4^ cells/well in triplicate and incubated for 24 h. Later, the hydrogel samples (GelMA, GelMA/AgNP, GelMA/HA, and GelMA/HYA) were separately placed on each well in sterile conditions and incubated for 24 h. After the incubation time, the treatment medium in the wells was replaced with 100 μL of fresh culture medium. Then, 10 μL of MTT solution (5 mg/mL in PBS) was added to all wells. After the microplates were additionally incubated for 4 h, the medium was carefully discharged, and 100 μL of DMSO was added to each well to solubilize the formazan crystals formed by viable cells. Then, the microplates were shaken at 150 rpm for 5 min. The absorbance (Abs) was measured at 540 nm using a spectrophotometer (ThermoScientific, Multiskan FC, Waltham, MA, USA). The cells not treated with the hydrogelwere used as a control group. The cells were photographed before the addition of MTT. All experiments were repeated three times and the results are expressed as the percentage of cell viability according to the following equation [42].

Cell Viability% = (Mean Abs of treated cells/Mean Abs of control or untreated cells) × 100

### 2.7. Antimicrobial Activities of GelMA Hydrogels

*Enterococcus faecalis* ATCC 29212, *Staphylococcus aureus* ATCC 6538/P, *Escherichia coli* ATCC 35218, *Pseudomonas aeruginosa* ATCC 27853, and *Candida albicans* ATCC 10239 strains were used for the antimicrobial activity test in the study. *Escherichia coli*, *Staphylococcus aureus*, and *Pseudomonas aeruginosa* were incubated for 24 h at 37 °C in Müller Hinton Broth. However, *Enterococcus faecalis* was incubated at 35 °C and *Candida albicans* at 25 °C for 48 h in Müller Hinton Broth.

The incubated microorganisms were adjusted to the 0.5 Mc Farland standard. Agar well diffusion experiments were conducted in Petri dishes with a diameter of 90 mm containing Mueller Hinton Agar to a depth of about 4 mm to determine the antimicrobial activity. For this purpose, 200 µL of each microorganism was inoculated separately into 25 mL Mueller Hinton Agars and poured into petri dishes. After the solidification of the Petri dishes, wells were drilled on the agar using a sterile cork drill with a diameter of 6 mm, and 50 µL of the tested hydrogels was dripped into these wells. The prepared Petri dishes were kept at 4 °C for 24 h to diffuse the hydrogels. After that, the Petri dishes were left incubating for 24 and 48 h under an appropriate incubation temperature for each microorganism. All tests were performed in two copies under aseptic conditions. Microbial growth was determined by measuring the diameter of the inhibition site in millimeters [43,44,45].

### 2.8. Statistical Analysis

The results of the antimicrobial activity assays are expressed as mean ± standard error. The results were analyzed using the Statistical Package for the Social Sciences (SPSS) software version 22.0 for Windows, and differences between groups were determined using One-Way ANOVA and Duncan’s multiple range test. The significant difference value was determined as *p* ≤ 0.05.

## 3. Results

### 3.1. GelMA Characterization

#### Determination of the Degree of Substitution

The TNBS (2,4,6-trinitrobenzene-1-sulfonic acid) method was used to determine the degree of GelMA substitution. In the TNBS method, a calibration graph was prepared with glycine. Depending on the obtained graph, the degree of substitution of the synthesized GelMA was determined. In the TNBS analysis, glycine solutions in different concentrations were prepared, while the calibration graph was prepared with glycine. In total, 0.5 mL of 0.01% TNBS solution was added to 0.5 mL of standard solution. The obtained mixture was incubated at 37 °C for 2 h. The results were evaluated by reading the absorbance at 335 nm and the degree of substitution was calculated as 82.27 ± 0.05% according to the TNBS analysis.

### 3.2. Injectable GelMA Hydrogels and GelMA Biocomposite Hydrogels Synthesis

**Figure 2:**
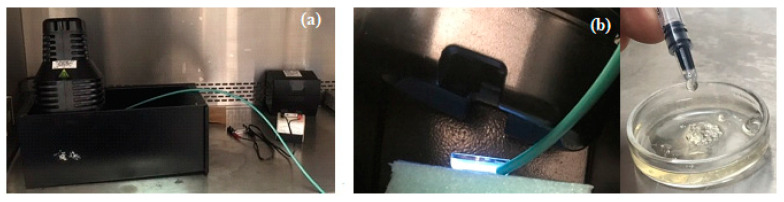
(**a**) GelMA hydrogel synthesis devices and (**b**) injectable GelMA hydrogel synthesis.

Injectable GelMA hydrogels were synthesized under sterile conditions in a laminar flow cabinet, with the assembly given in Figure 2a. The Petri dishes and injectable form in which the hydrogel is synthesized are given in Figure 2b.

### 3.3. GelMA and GelMA Hydrogel Characterization

#### 3.3.1. FTIR Characterization of GelMA

In this study, O-H and N-H stretching peaks at 3280 cm^−1^, saturated C-H stretching peaks at 2940 cm^−1^, amide I peaks at 1630 cm^−1^, and amide II peaks at 1520 cm^−1^ were observed in the synthesized samples of GelMA. The presence of these peaks shows that the synthesis of GelMA from gelatin occurred at a large rate.

#### 3.3.2. H-NMR Characterization of GelMA

**Figure 3:**
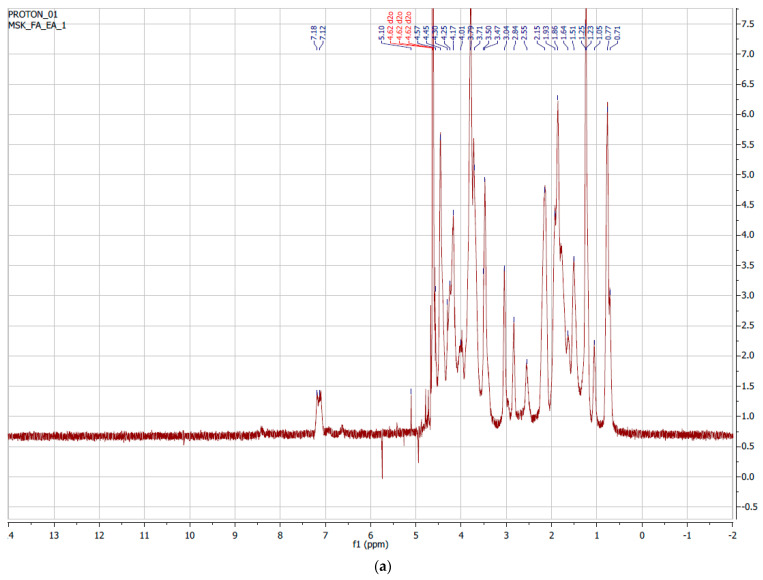

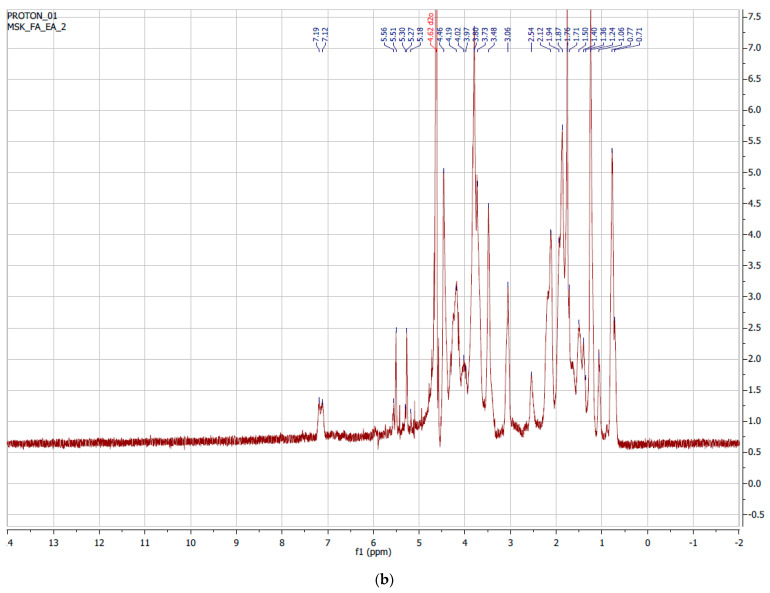
^1^H-NMR spectrum of (**a**) gelatin Type B and (**b**) GelMA.

Nuclear Magnetic Resonance (NMR) measurements of the pure gelatin of the synthesized GelMAs were performed. The obtained NMR data were examined and the degree of methacrylation was determined. Figure 3a shows the ^1^H-NMR spectra of pure Type B gelatin. The signal from the methylene group of lysine amino acid in the structure of gelatin was observed between 2.8 and 2.95 ppm. In Figure 3b, it can be observed that there was no signal from lysine seen at 2.8–2.95 ppm. It was observed that there was a higher yield than the GelMA synthesized in a single buffer medium. At the same time, it was observed that the signal from lysine between 2.8 and 2.95 ppm decreased or disappeared, and a signal from methacrylate between 5.4 and 5.6 ppm occurred. When we examine the signals in the spectra, it can be said that GelMAs were successfully synthesized from gelatin. The degree of methacrylation was found to be greater than 95% after examining the NMR peak regions in the synthesis of GelMA from gelatin. The equation below was used for this calculation.

$$\%\;\mathrm{The}\;\mathrm{degree}\;\mathrm{of}\;\mathrm{methacrylation}\;(\mathrm{DM})=(\frac{peak\;area(GELMA\;lysine\;methylene)}{peak\;area(lysine\;methylene\;of\;unmodified\;gelatin)})\times 100$$

#### 3.3.3. SEM Characterization of GelMA Hydrogel

**Figure 4:**
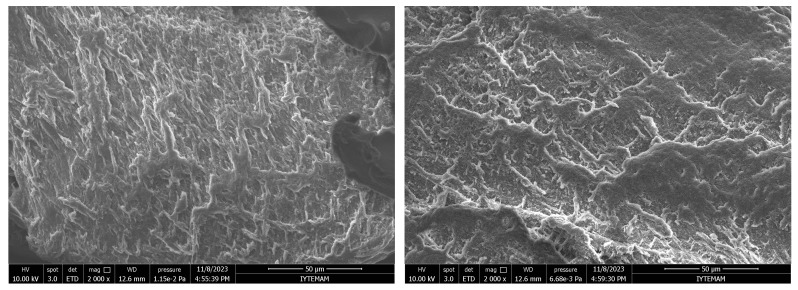

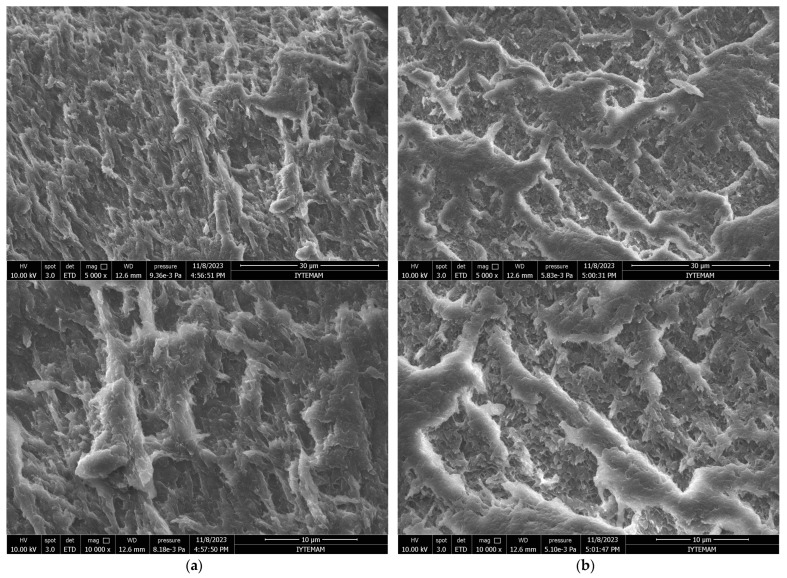
SEM micrographs of GelMA hydrogels: (**a**) surface and (**b**) cross-section (2000×, 5000×, and 10,000× Magnifications from top to bottom).

As can be seen in the Scanning Electron Microscope (SEM) images, all synthesized hydrogels exhibited a highly interconnected porous structure (Figure 4). The porous structure was monitored in such a way that smaller pores were separated by a thin wall. This proves that hydrogels are suitable for the survival of cells and nutrient exchange.

#### 3.3.4. Swelling Behavior of GelMA Hydrogels

The synthesized GelMA hydrogels were dried in a vacuum oven at 37 °C for the swelling experiments. The GelMA hydrogels were dried until they had constant weight. The swelling behavior of these hydrogels was studied at pH = 7.4 PBS buffer.

**Figure 5:**
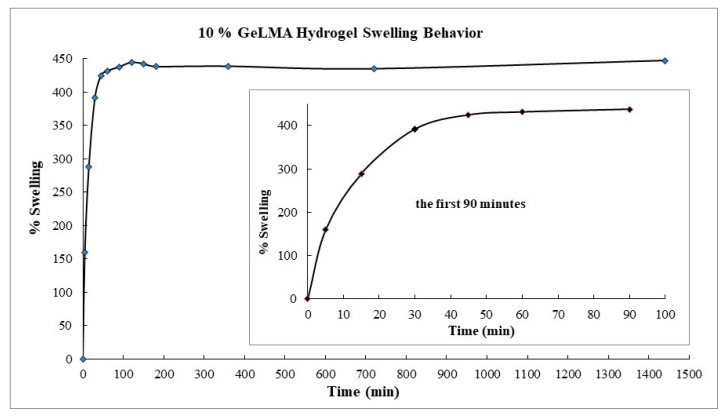
Swelling behavior of GelMA hydrogels.

The swelling experiments were conducted in a hot water bath at 37 °C at a churning rate of 100 cpm (Figure 5).

When the swelling behavior of the GelMA hydrogels was examined, it was observed that they swelled by 160% in the fifth minute. It was observed that this occurred at 288% in the fifteenth minute. The swelling reached 430% in the ninetieth minute, and then it was seen that the swelling did not change. In short, it was concluded that the hydrogel with a low GelMA content swelled more, and the swelling would decrease as the amount of GelMA in it increased. From here, it was concluded that hydrogels containing a small amount of GelMA had wider pores and swelled more comfortably.

#### 3.3.5. Biodegradation Behavior of GelMA Hydrogel

**Figure 6:**
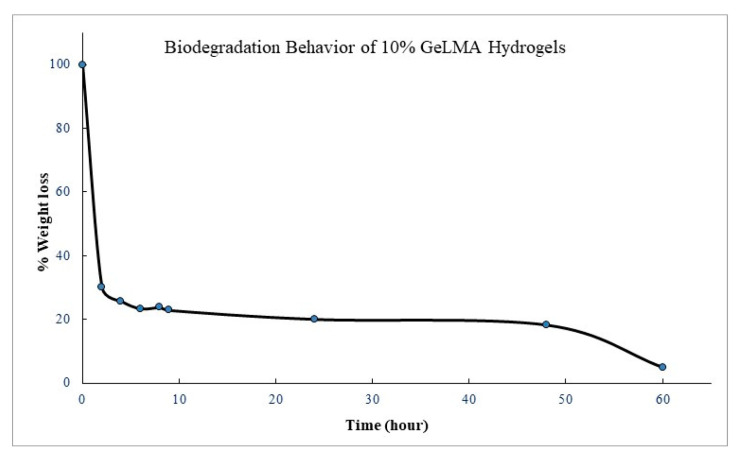
Biodegradation behavior of GelMA hydrogels.

After the GelMA hydrogels were synthesized, hydrolytic degradation studies were performed. Degradation studies were carried out by taking measurements at regular intervals in a water bath with a shaking temperature of 100 cpm at 37 °C at a buffer pH = 7.4 PBS. The measurements were continued until the degradation of the hydrogel was completed. When the biodegradation trials of the GelMA hydrogels were examined, it was observed that the biodegradation reached 97% at the end of approximately 60 h in the hydrolytic degradation of 10% GeLMA hydrogels. It was observed that the biodegradation of hydrogels reached 70% in the second hour. But then the rate of degradation continued to slow down. The experiment was continued until the degradation of the hydrogels was completed (Figure 6). The biodegradations of the GelMA biocomposite hydrogels were not significantly affected by the presence of HYA, HA, and AgNPs, which was interpreted as because of the absence of chemical interaction between GelMA and other constituents.

### 3.4. AgNP Synthesis (Green Synthesis)

**Figure 7:**
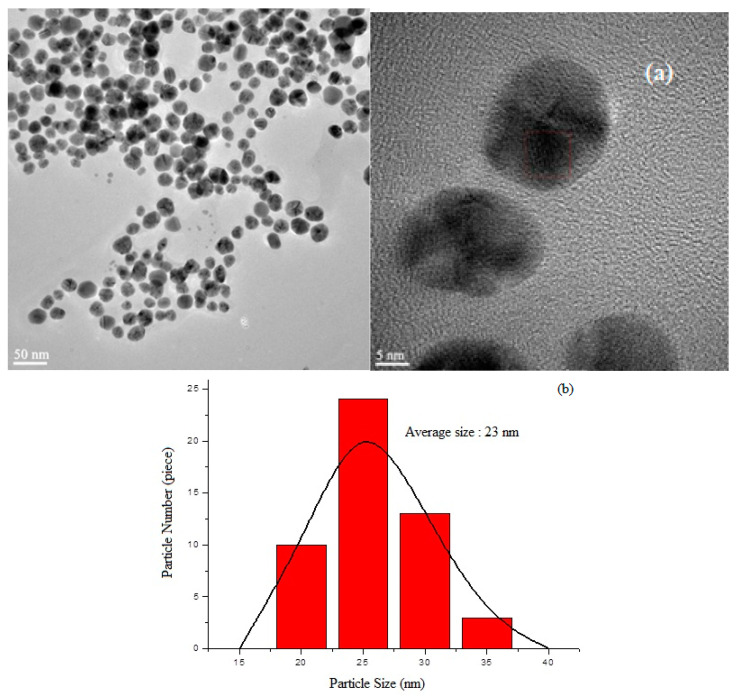

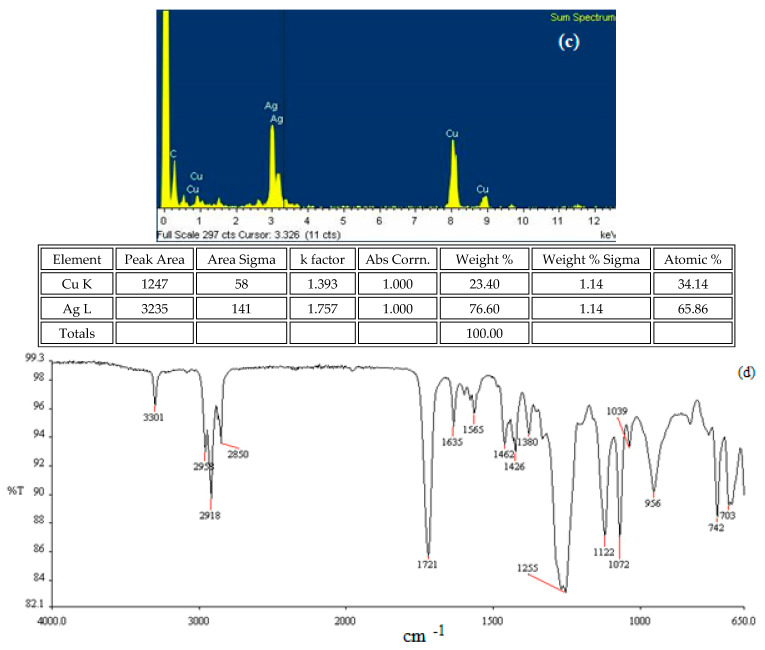
Synthesized AgNPs: (**a**) TEM photos; (**b**) particle size distribution; (**c**) EDS analysis; (**d**) and FTIR spectrums of synthesized AgNPs.

The findings obtained in the research are given in detail below. A High-Resolution Transmission Electron Microscopy (HRTEM) image of AgNPs synthesized with local plant leaf extract in accordance with the environmentalist synthesis approach is given in Figure 7a. The particle size distribution of the AgNPs is shown in Figure 7b. Under the given synthesis conditions, it was determined that the AgNPs had a size distribution of an average diameter of 23 ± 7.21 nm. The Energy Dispersive Spectrum (EDS) Analysis of the synthesized AgNPs and the FT-IR spectrum of the AgNPs are given in Figure 7c and 7d, respectively.

The tentative frequency assignments of the FT-IR spectrum of the AgNPs were evaluated. The bending vibration at a wave number of 1721 cm^−1^ indicates the stretching vibration of the carbonyl of the free keto group effective in the formation of the AgNPs. The tensile vibration at a wave number of 3301 cm^−1^ was greatly due to the OH stretching of the H-bonded OH groups, and the stretching vibration at a wave number of 1225 cm^−1^ was due to aromatic groups, while that at 1255 cm^−1^ was due to the C-O stretching (ethers)/C-N stretching (amines) [46,47].

By analyzing the TEM-associated technique Energy-dispersive X-ray (EDS spectrum), the presence of AgNPs was confirmed by a distinct peak at 3.0 keV, and silver was about 65%. Another moderate signal of cupper was found, greatly as a result of the preparation of the sample using a copper grid. The reductive agent extract caused the appearance of other weak signals of oxygen, sulfur, and silisium.

### 3.5. The Effects of Hydrogels on Cell Viability

Cell culture studies of the synthesized GelMA hydrogels were performed. The effects of the hydrogels on cell viability were studied in the cell culture studies. L929 fibroblast cells were used in cell interaction studies. Photographs of the cells were taken 24 h after the uploads and cell viability studies were performed.

**Figure 8:**
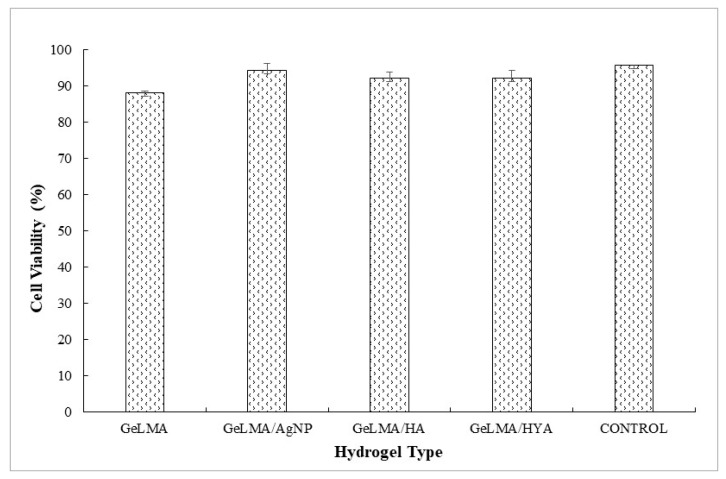
Cell viability of GelMA hydrogels.

The L929 fibroblast cells interacted with injectable GelMA hydrogels. Cell viability was measured by an MTT test at the end of 24 h. There was no significant difference (*p* > 0.05). It was observed that the synthesized injectable gel hydrogels were similar to the cell viability control group (Figure 8).

### 3.6. Antimicrobial Activities of GelMA Hydrogels

**Figure 9:**
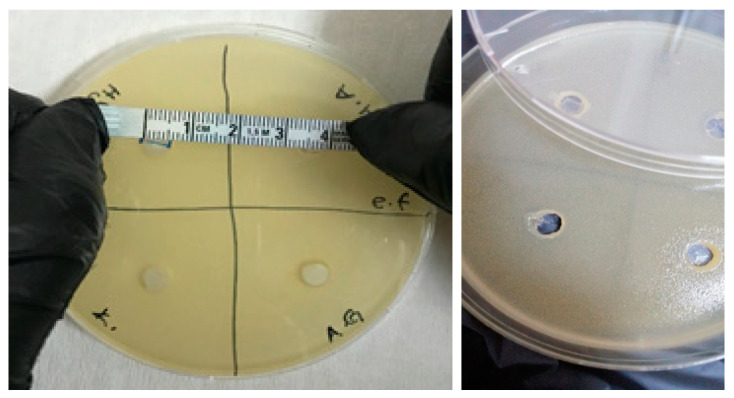
Photos of *Enterococcus faecalis* (ATCC 29212 strain) after 24 h incubation (the highest diameter is seen in GelMA/HYA hydrogel).

**Figure 10:**
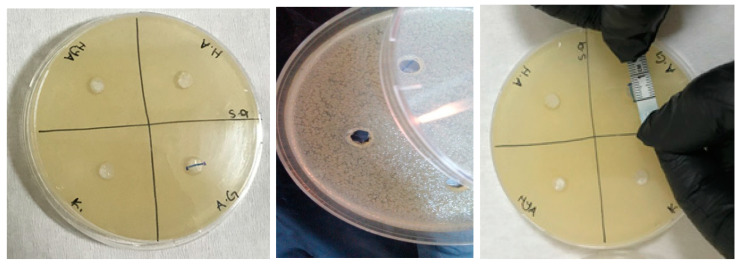
Photos of *Staphylococcus aureus* (ATCC 6538/P strain) after 24 h incubation (the highest diameter is seen in GelMA/AgNP hydrogel).

**Figure 11:**
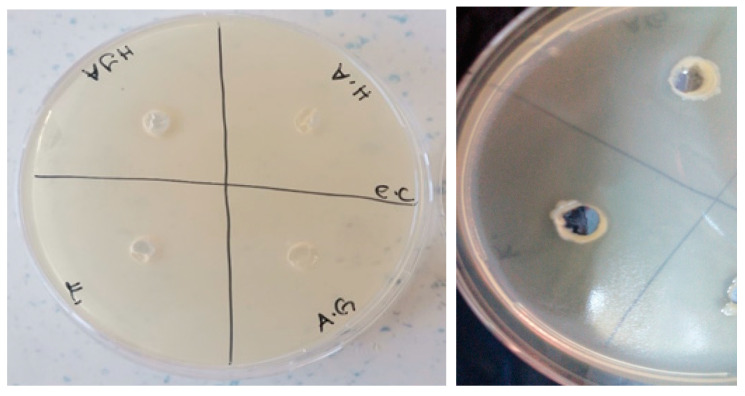
Photos of *Escherichia coli* (ATCC 35218 strain) after 24 h incubation (the highest diameter is seen in GelMA/AgNP hydrogel).

**Figure 12:**
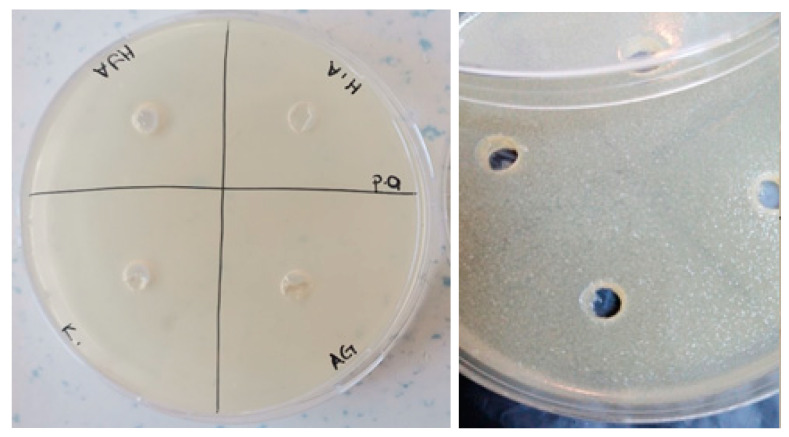
Photos of *Pseudomonas aeruginosa* (ATCC 27853strain) after 24 h incubation (the highest diameter is seen in GelMA/AgNP hydrogel).

**Figure 13:**
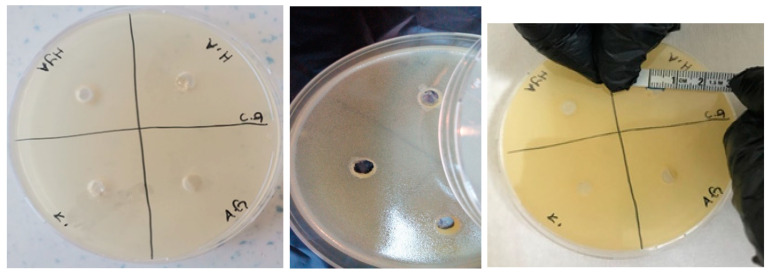
Photos of *Candida albicans* (ATCC 10239 strain) after 24 h incubation (the highest diameter is seen in GelMA/HA hydrogel).

The antimicrobial activities of the synthesized gels were evaluated against both Gram-positive (*Enterococcus faecalis* and *Staphylococcus aureus*) and Gram-negative (*Escherichia coli* and *Pseudomonas aeruginosa*) bacteria and fungi (*Candida albicans*) using the agar well diffusion method. The results of the antimicrobial activities of hydrogels are given in Table 1. Accordingly, the antimicrobial activity was observed to not change after 24 and 48 h of incubation (Figure 9, Figure 10, Figure 11, Figure 12 and Figure 13).

The GelMA/HYA was the most effective hydrogel against *Enterococcus faecalis*, with a zone diameter of 5.5 ± 0.35mm. The most effective hydrogel against *Staphylococcus aureus* (7.5 ± 0.35 mm) and *Escherichia coli* (6 ± 0.00 mm) was GelMA/AgNP. GelMA/AgNP and GelMA/HA were the most effective hydrogels against *Pseudomonas aeruginosa*, with zone diameters of 7 ± 0.00 mm and 6.25 ± 0.18 mm, respectively.

In addition, GelMA/HA was also the most effective hydrogel against *Candida albicans*, with a zone diameter of 6.75 ± 0.18 mm. When the antibacterial results were evaluated together, it appeared that GelMA/AgNP had strong antibacterial activity among the hydrogels.

It was observed that the GelMA/HA hydrogel, which was the most effective against *Candida albicans*, and the GelMA/HYA hydrogel, which was the most effective against *Enterococcus faecalis*, also showed antimicrobial properties against the other microorganisms tested.

## 4. Discussion

In this study, we aimed to evaluate the antimicrobial effectiveness of the production of bioactive and biomimetic injectable tissue scaffolds under in vitro conditions by taking advantage of the developments obtained in the field of biomaterials and tissue engineering in the practice of regenerative endodontics. A recent systematic review showed that 79% of failed regenerative clinical cases are caused by persistent infection, which means that controlling the bacterial load in the root canal system is the most critical step in regenerative endodontic procedures [48,49]. In regenerative endodontics applications, it is recommended to use triple antibiotic pat (TAP) or calcium hydroxide as an intra-channel drug between sessions to ensure the decontamination of root canals [50].

However, various disadvantages have also been reported when using TAP, such as cytotoxicity against stem cells, the risk of tooth discoloration, the development of drug resistance, and the difficulty of removal from the canal [12,51]. The antimicrobial activity of antibiotic-loaded chitosan hydrogels was studied using *E. faecalis*. Chitosan hydrogels loaded with dual antibiotic pat were reported to be successful in terms of antibacterial activity and cell viability. Although chitosan has an antibacterial property, it has been effective against *Enterococus faecalis*, which is a source of stubborn infections, along with antibiotic loading. But the use of antibiotics is trying to be reduced due to their antibiotic resistance and the presence of residual antibiotics remaining in the canal walls [52]. For this reason, alternative applications to the use of antibiotics have been studied.

Injectable hydrogel-based materials have been used as tissue scaffolds for regenerative endodontic procedures [53]. Gelatin methacryloyl (GelMA) hydrogels are a widely used material for biomedical applications due to their modifiable, suitable, and adaptable physical and biological properties. In addition, it is known that antimicrobial properties can be acquired by adding functional groups with natural antimicrobial abilities [54]. When studies in the literature are examined, it is known that silver nanoparticles (AgNPs) have strong antimicrobial activities [55]. As a special antimicrobial substance, silver ions have broad-spectrum antimicrobial activity against microorganisms, especially some resistant species. Recent studies have shown that the use of silver nanoparticles as an antibiotic agent for antibacterial materials is a promising approach for medical applications. Silver nanoparticles can penetrate the cell wall or accumulate on the cell wall and affect electrolyte and metabolite transport, cause interactions with membrane proteins, activate signaling pathways, and prevent cell proliferation and the contraction or separation of the cytoplasmic membrane from the cell wall [56,57,58,59]. Wang et al. [34] tested the antibacterial activity of AgNP-loaded GelMA hydrogels and reported that the synthesized hydrogel showed a good antibacterial effect on *E. coli* and *S. auereus* in their studies. In this study, it was also found that the inhibition zone formed by the silver-nanoparticle-laden hydrogel on *E. coli* and *S. aureus* was the largest.

Hydroxyapatite (HA) is widely used in medical research because the mineral has a similar component to the human skeleton and is known to have exceptional biocompatibility, bioactivity, and chemical stability [60]. Also, Ragap et al. [30]., in their study, reported that hydroxyapatite nanoparticles are active against the most common Gram-positive and Gram-negative bacteria and are an advantageous material for clinical applications. In this study, it was observed that HA had an effect on *C. albicans* and inhibited growth on other microorganisms to different degrees.

When studies with hyaluronic acid (HYA) are examined, it is seen that the prepared composites can prevent bacterial adhesion to the substrate depending on the concentration and are active when treated as a pure substance [61]. In addition, the bacteriostatic effects of hyaluronic acid on oral and non-oral microorganisms have been investigated in dental surgery, and it has been observed that prepared hyaluronic-acid-containing gels and solutions show bacteriostatic effects [62].

When looking at the results of the HYA-loaded hydrogel, it was observed that it showed the highest effectiveness on E faecalis, but also had an antimicrobial effect on the microorganisms used in the study. When looking at the cell wall structures of Gram (+)-positive and Gram (−)-negative bacteria, Gram (+)-positive bacteria have a thicker peptidoglycan cell wall, while Gram (−)-negative bacteria have thinner cell walls. In addition, their lipopolysaccharide (LPS) layer makes Gram (−)-negative bacteria more resistant. In Gram (+)-positive bacteria, the hydrogel can affect the cell by disrupting the structure of the peptidoglycan layer, but in Gram (−)-negative bacteria, the LPS layer can limit the effect of the hydrogel [63]. Some studies have suggested that Gram-positive bacteria are more sensitive to Ag+ than Gram-negative bacteria. Hydrogels can strongly adhere to the negative-charge LPS layer located on the outer membrane of Gram-negative bacteria, but it cannot reach the peptidoglycan layer, while it will bind to the peptidoglycan layer of Gram-positive bacteria and damage it. According to this argument, Ag+ is more likely to damage a Gram (+)-positive cell [64].

As a result, when the microorganisms were evaluated according to their cell wall structures, it was observed that the synthesized HYA-loaded and AgNP-loaded hydrogels were more effective against the Gram (+)-bacteria *E. faecalis* and *S. aureus*, respectively, creating the highest inhibition zones.

It is very important to determine cytotoxicity in vitro to confirm the use of biocomposite injectable hydrogel systems synthesized for the regeneration of the pulp–dentin complex. There are many studies in the literature in which the cytotoxicity of GELMA has been evaluated by preparing it at different concentrations, and the result that there are no cytotoxic effects has been reported in these studies [65,66,67,68]. The biocomposite in injectable form synthesized within the scope of this study does not show a cytotoxic effect on hydrogels.

## 5. Conclusions

In this study, injectable biocomposite hydrogels exhibiting effective antimicrobial activity and non-cytotoxic behavior were successfully synthesized. This is also promising in clinical applications in regenerative endodontic procedures from hydrogels, which are proposed based on the collected data. The GelMA hydrogel loaded with hyaluronic acid showed the highest efficacy on entrococcus faecalis, one of the stubborn bacteria in the root canal. The GelMA hydrogel loaded with hydroxyapatite also showed a significant effect on candida albicans, which is another bacteria responsible for stubborn infections in the root canal. It is very important to investigate the effectiveness of this new injectable system with animal studies before further clinical trials (for example, the periapical disease model in sheep).

## Figures and Tables

**Table 1:** Antimicrobial activity of GelMA, GelMA/AgNP, GelMA/HA, and GelMA/HYA against selected pathogens by agar diffusion method.

	Inhibition Zone Diameters (mm)							
	GelMA		GelMA/AgNP		GelMA/HA		GelMA/HYA	
Microorganism Type	24 h *	48 h *	24 h *	48 h *	24 h *	48 h *	24 h *	48 h *
*Enterococcus faecalis* ATCC 29212	Bottom of sample ** 2 ± 0.00 ^c^	Bottom of sample ** 2 ± 0.00 ^c^	Bottom of sample ** 4 ± 0.00 ^a,b^	Bottom of sample ** 4 ± 0.00 ^a,b^	Bottom of sample ** 3 ± 0.00 ^b,c^	Bottom of sample ** 3 ± 0.00 ^b,c^	Bottom of sample ** 5.5 ± 0.35 ^a^	Bottom of sample ** 5.5 ± 0.35 ^a^
*Staphylococcus aureus* ATCC 6538/P	Bottom of sample ** 3.75 ± 0.18 ^b^	Bottom of sample ** 3.75 ± 0.18 ^b^	6 + 1.5 (Well diameter + zone) *** ±0.35 ^a^	6 + 1.5 (Well diameter + zone) *** ±0.35 ^a^	Bottom of sample ** 5.5 ± 0.35 ^a,b^	Bottom of sample ** 5.5 ± 0.35 ^a,b^	Bottom of sample ** 5 ± 0.00 ^b^	Bottom of sample ** 5 ± 0.00 ^b^
*Escherichia coli* ATCC 35218	Bottom of sample ** 3 ± 0.00 ^d^	Bottom of sample ** 3 ± 0.00 ^d^	Bottom of sample ** 6 ± 0.00 ^a^	Bottom of sample ** 6 ± 0.00 ^a^	Bottom of sample ** 5.25 ± 0.18 ^a,b^	Bottom of sample ** 5 ± 0.00 ^b,c^	Bottom of sample ** 4.25 ± 0.18 ^c^	Bottom of sample ** 4.25 ± 0.18 ^c^
*Pseudomonas aeruginosa* ATCC 27853	Bottom of sample ** 2.5 ± 0.35 ^b^	Bottom of sample ** 2.5 ± 0.35 ^b^	6 + 1 (Well diameter + zone) *** ±0.00 ^a^	6 + 1 (Well diameter + zone) *** ±0.00 ^a^	6+0.25 (Well diameter + zone) *** ±0.18 ^a^	6 + 0.25 (Well diameter + zone) *** ±0.18 ^a^	Bottom of sample ** 3.5 ± 0.35 ^b^	Bottom of sample ** 3.5 ± 0.35 ^b^
*Candida albicans* ATCC 10239	Bottom of sample ** 1.75 ± 0.18 ^b^	Bottom of sample ** 1.75 ± 0.18 ^b^	Bottom of sample ** 2.5 ± 0.35 ±0.00 ^b^	Bottom of sample ** 2.75 ± 0.18 ^b^	6 + 0.75 (Well diameter + zone) *** ±0.18 ^a^	6 + 0.75 (Well diameter + zone) *** ±0.18 ^a^	Bottom of sample ** 4 ± 0.70 ^a,b^	Bottom of sample ** 4 ± 0.70 ^a,b^

* The data in the table are the 24 and 48 h results of the study conducted by agar well diffusion method in two repetitions. ** “Bottom of sample” shows the diameters of the non-breeding zones observed on the Petri base when the hydrogel was removed, the well diameters are 6 mm. *** “Well diameter + Zone” states that there is no reproduction in the drilled well and that reproduction is prevented at the specified diameter in the agar. Data are presented as the mean ± SE, different superscript letters in the same line denote significant differences (Duncan’s range test, *p* ≤ 0.05). According to the Duncan analysis results, the hydrogel with the most effect is designated with the letter “a” and is named alphabetically in order of effect. In statistical analysis, each microorganism was evaluated on its own.

## Data Availability

Data are contained within the article.
